# Macimorelin Acetate for the Diagnosis of Childhood-onset Growth Hormone Deficiency

**DOI:** 10.17925/EE.2022.18.2.84

**Published:** 2022-09-08

**Authors:** Rohan K Henry

**Affiliations:** Section of Endocrinology, Department of Pediatrics, Nationwide Children’s Hospital, The Ohio State University College of Medicine Columbus, OH, USA

**Keywords:** Childhood-onset growth hormone deficiency, growth hormone deficiency, growth hormone, macimorelin acetate, insulin tolerance test, provocative test

## Abstract

Growth hormone provocation testing forms the cornerstone of the diagnosis of childhood growth hormone deficiency in clinical practice. Despite the widespread use of these tests, various criticisms have been levelled against them, such as the labour-intensive nature of the tests, their potential for serious adverse effects and their questionable reproducibility. Macimorelin acetate, a ghrelin mimetic approved for the diagnosis of adult growth hormone deficiency, could serve an unmet need in the diagnosis of childhood-onset growth hormone deficiency based on its good tolerability and benign side effect profile.

In a 1958 article titled “Treatment of a pituitary dwarf with human growth hormone”, the merits of human growth hormone (GH) as a treatment for GH deficiency (GHD) were outlined by Raben.^1^ Beyond its use as a treatment for short stature, GH can be used for other manifestations associated with a GH-deficient state. Therefore, establishing a diagnosis of GHD is an important step in determining who qualifies to receive therapy.

Recurrent cycles throughout a 24-hour period, known as an ultradian rhythm, characterize GH production. Due to this rhythmicity, provocative testing remains the cornerstone for diagnosing GHD in many countries, with the exception of Australia.^2^ These tests have evolved, and the level below which GHD is diagnosed has also changed.^3^ Although there is arguably no true gold standard testing for GHD, the insulin tolerance test (ITT) is often seen as a reference standard. Its former use was widespread; however, it has fallen out of favour with many clinicians as it is labour intensive and carries the risk of hypoglycaemia, seizures and even death.^4^ Based on its potential for serious morbidity and even mortality, the search for agents with a more tolerable adverse effect profile led to the discovery of other tests. Although the mechanism responsible for the production of GH during provocation testing may be debatable for an agent such as glucagon, these tests are still used worldwide.^5,6^ Agents used for testing, which include levodopa (L-DOPA), glucagon, arginine and clonidine, have been relied on to support auxological data in the presence of historical findings to rule out GHD in a subset of patients from the general pool presenting for growth evaluation. These important findings include a decrease in a patient’s height standard deviation score not in keeping with an established familial pattern or a change in the patient’s height below -2 standard deviation score and/or a change in his/her height velocity pattern.^7^ Other supportive data that should heighten the suspicion for GHD include a delayed bone age and a reduced level of insulinlike growth factor 1, though the latter should be interpreted with caution based on nutrition status and in children under 5 years old. These parameters as criteria to perform provocation testing cannot be overemphasized, as careful patient selection will increase the predictive value of testing considering the inherent test weaknesses discussed below.

Agents used in provocation testing may be associated with possible adverse effects. Therefore, clinicians need to be aware of the adverse effect profile of each medication. Hypotension can be associated with L-DOPA and clonidine, whereas sleepiness can be attributed to clonidine use. Nausea can be encountered with arginine, L-DOPA testing and glucagon administration, whereas hypoglycaemia is a well-recognized adverse effect seen with glucagon use.^8^ GH levels derived from testing can be impacted by sex steroid priming in peri-pubertal aged children, a point to consider as patients with constitutional delay of growth and puberty may present with growth concerns.^9^ In addition, various criticisms have been levelled against the use of these tests for the diagnosis of GHD. These include the arbitrariness of a single cutoff level below which GHD is diagnosed, even though there is differential stimulation of GH production by different agents, and these tests have questionable reproducibility. These issues are also compounded as tests have limited discriminating ability to differentiate children with GHD from children with short stature but normal growth.^10^ Based on the combination of the serious adverse effect profile associated with some agents and the limitations cited for provocative tests as a whole, there is currently an unmet testing need for diagnosing childhood GHD. Hence, the development of alternative methods for diagnosing childhood GHD remains crucial, and such testing may potentially be transformative.

The ideal agent used for GH provocative testing should be reliable in eliciting a response, not labour intensive to administer and, ultimately, safe. Moreover, it should be able to discriminate short children with normal growth from those with GHD, and a new potential test with good sensitivity and specificity in diagnosing paediatric GHD, unlike the current tests, which differentially elicit GH production, is needed.

Macimorelin acetate, an orally administered ghrelin mimetic, has been studied for diagnosing GHD in adults, and testing has begun with this in paediatric populations.^2^ In adults, macimorelin acetate has been validated against the ITT, and the data showed that it is safe and has good tolerability, with the most frequent side effect being dysgeusia, which is usually both mild and transient; it also has good accuracy and reproducibility compared with the ITT. In a phase III open-labelled, randomized multicentre, two-way crossover study conducted in the USA and Europe, macimorelin acetate versus the ITT were compared in 33 adults (Validation of macimorelin as a test for adult growth hormone deficiency; ClinicalTrials.gov identifier: NCT02558829).^12^ Using a GH cutoff of 5.1 ng/mL for both tests, test sensitivity and specificity were 92% and 96%, respectively. A cutoff of 5.1 ng/mL provided a good trade-off between sensitivity and specificity compared with a cutoff of 2.8 ng/mL, for which the sensitivity was 87% and specificity was 96%.^12^

In the paediatric population, an open-label group comparison, dose-escalation trial was performed to study the safety, tolerability, pharmacokinetics and pharmacodynamics of macimorelin acetate.^13^ In this study of 24 children with suspected GHD aged between 2 and 18 years, macimorelin acetate was administered to sequential patient cohorts of eight patients who received ascending single doses of 0.25, 0.5 and 1 mg/kg. Macimorelin provocation testing was performed between 2 standard GH stimulation tests separated by a recovery period of 7–28 days between tests. Primary endpoints were safety and tolerability, and secondary endpoints were pharmacokinetics and pharmacodynamics. There was a dose-dependent increase in plasma concentration with maximum levels observed between 15 and 120 minutes. Based on the receiver operating characteristic and sensitivity analysis, the strongest characteristics were observed at the highest dosing (sensitivity 100%, specificity 80%; area under the receiver operating characteristic curve 93%). Regarding safety and tolerability, however, there were a total of 158 adverse events and treatment-emergent adverse events combined, although none was caused by macimorelin acetate.^13^

As macimorelin acetate is administered orally (although it may still require an infusion centre or a hospital setting and intravenous access for blood draws), based on the safety and tolerability data, the acuity level for macimorelin testing could be lower, with fewer staff members required compared with agents with more serious side effect profiles.

Moreover, macimorelin acetate testing used as a single agent could be of shorter duration compared with many existing protocols for current agents, which require two independent agents, often sequentially administered under protocols that are invariably often at least 3 hours long. As such, although testing may be of a duration similar to that of an individual test such as clonidine testing (90 minutes) based on some protocols, if used as a single test, it could be less time-consuming. To further this point, in the paediatric study mentioned above, peak GH levels occurred between 15 and 60 minutes; hence, there is the possibility of even shorter testing durations.^2^ Despite the many positive points outlined, and similarly to the currently administered agents, the use of macimorelin will not obviate the need for sex steroid priming.

Considering the aforementioned points, macimorelin acetate could be impactful in the diagnosis of childhood-onset GHD. However, ongoing studies will be needed to ensure that its use is generalizable to diverse paediatric populations, as factors such as body mass index may affect GH levels derived from provocation testing, and the effects of sex and age on testing will need further exploration.
